# A minimal peach type II chlorophyll *a/b*-binding protein promoter retains tissue-specificity and light regulation in tomato

**DOI:** 10.1186/1472-6750-7-47

**Published:** 2007-08-14

**Authors:** Carole L Bassett, Ann M Callahan, Timothy S Artlip, Ralph Scorza, Chinnathambi Srinivasan

**Affiliations:** 1 US Department of Agriculture, the Agricultural Research Service, Appalachian Fruit Research Station, 2217 Wiltshire Road, Kearneysville, West Virginia, 25430, USA

## Abstract

**Background:**

Promoters with tissue-specificity are desirable to drive expression of transgenes in crops to avoid accumulation of foreign proteins in edible tissues/organs. Several photosynthetic promoters have been shown to be strong regulators of expression of transgenes in light-responsive tissues and would be good candidates for leaf and immature fruit tissue-specificity, if expression in the mature fruit were minimized.

**Results:**

A minimal peach chlorophyll *a/b*-binding protein gene (Lhcb2*Pp1) promoter (Cab19) was isolated and fused to an *uid*A (β-glucuronidase [GUS]) gene containing the PIV2 intron. A control vector carrying an enhanced *mas*35S CaMV promoter fused to *uid*A was also constructed. Two different orientations of the Cab19::GUS fusion relative to the left T-DNA border of the binary vector were transformed into tomato. Ten independent regenerants of each construct and an untransformed control line were assessed both qualitatively and quantitatively for GUS expression in leaves, fruit and flowers, and quantitatively in roots.

**Conclusion:**

The minimal CAB19 promoter conferred GUS activity primarily in leaves and green fruit, as well as in response to light. GUS activity in the leaves of both Cab19 constructs averaged about 2/3 that observed with *mas*35S::GUS controls. Surprisingly, GUS activity in transgenic green fruit was considerably higher than leaves for all promoter constructs; however, in red, ripe fruit activities were much lower for the Cab19 promoter constructs than the *mas*35S::GUS. Although GUS activity was readily detectable in flowers and roots of *mas*35S::GUStransgenic plants, little activity was observed in plants carrying the Cab19 promoter constructs. In addition, the light-inducibility of the Cab19::GUS constructs indicated that all the requisite *cis*-elements for light responsiveness were contained on the Cab19 fragment. The minimal Cab19 promoter retains both tissue-specificity and light regulation and can be used to drive expression of foreign genes with minimal activity in mature, edible fruit.

## Background

Introduction of foreign DNA into plants via *Agrobacterium *transformation has been well documented [1,2]. In most cases binary vectors containing T-DNA (transfer DNA) are used, and transformed cells are selected by expressing vector genes encoding resistance to antibiotics. In addition to selective genes, many vectors carry a reporter gene whose expression can be conveniently monitored by enzyme activity (*uid*A = β-glucuronidase [GUS]), fluorescence (Green Fluorescent Protein [GFP]) or bioluminescence (luciferase). These reporter genes are often used to monitor regulation of expression controlled by promoters and/or other sequences influencing transcription. Indeed, mapping of regions in promoters responsible for activation or repression of gene expression is most conveniently done using a reporter gene [see for example [3,4]].

The most popular promoter used in plant transformation vectors is the cauliflower mosaic virus 35S (Cauliflower Mosaic Virus [CaMV] 35S) promoter and derivatives thereof. This promoter is considered to be powerful and constitutive, although some temporal and tissue-specificity has been reported [5,6]. It represents the most frequently used promoter in the development of first generation plant transformation vectors. Recently there has been renewed interest in developing second and third generation vectors improved in the control of transgene expression by incorporating tissue-specific promoters. Tissue-specific promoters offer more precise spatial control over target genes, allowing expression in one or a few tissues, but not in others. Of particular interest regarding consumer preference issues are those promoters which would allow expression of a transgene in a specific target tissue/organ while avoiding expression in edible plant tissue(s).

Tissue-specific promoters of interest in genetic engineering strategies include those associated with photosynthesis where expression is restricted to "green" tissues, and is considerably lower or absent in non-photosynthetic tissues like roots, mature flowers (excluding sepals) and mature fruit/nuts. Such promoters would be expected to promote relatively high levels of transgene expression in leaves, stems and in young floral buds and fruits. Several promoters of this type have already been described. Transformation of tomato plants with the tomato *RBCS3A *[Rubisco small subunit] gene promoter fused to GUS resulted in leaf-specific expression compared to green fruits [7]. In contrast, two other tomato *RBCS *promoters (*RBCS1 *and *RBCS2*) showed similar GUS activity in leaves and green fruits. Interestingly, transient expression of the *RBCS3A *construct showed no difference in expression of GUS between leaves and green fruit, implying that integration is necessary for specificity. A more recent study of *Agrobacterium*-mediated transgene expression in apple utilized Rubisco small subunit gene promoters from tomato (RBCS3C) and soybean (SRS1) to drive expression of GUS [8]. Expression in young apple seedling leaves was about 55–60% of that determined for a CaMV 35S-GUS control. Surprisingly, expression of the RBCS3C::GUS construct in leaves was independent of light, in contrast to previous observations of RBCS expression. Unfortunately, GUS activity could not be determined in fruits, as the apple seedlings had not reached reproductive maturity during the experiment; however, tissue-specificity was determined by measuring GUS activity in roots. For both RBCS constructs, GUS activity was substantially lower in roots compared to leaves. A study [9] using the strong photosynthetic promoter of PNZIP from *Pharbitis nil *(L.) to drive GUS expression reported high levels of expression in leaves and little expression in flowers and roots of transformed tobacco (*Nicotiana tabacum *L. 'NC89').

We are interested in identifying other photosynthetic promoters as potential candidates for regulating expression in leaves, but not in mature fruits. We previously [10,11] described the isolation and characterization of a type II chlorophyll *a/b*-binding protein (Cab) gene from peach (*Prunus persica *L. Batsch.). Expression of this Cab gene in developing leaves during the growing season followed patterns typical of developing leaves of herbaceous plants. Shaded leaves showed a profile similar to that of sun-exposed leaves, although Cab mRNA levels were in general lower in the shaded leaves. Of interest also is the observation that significant levels of Cab mRNA could be detected in very young fruit (up to 71 days after bloom) but not in mature, ripe peach fruits [[12]; Cab = pch108]. In the current study our objective was to use a minimal Cab promoter, which retained its native tissue- and light-specificity. To achieve this objective we identified the Lhcb2*Pp1 promoter, isolated a short (~500 bp) fragment, and fused it with the *uidA *reporter gene to monitor expression driven by the Cab promoter in a tomato model host.

## Results

### Promoter region of Lhcb2*.Pp1

**Figure 1:**
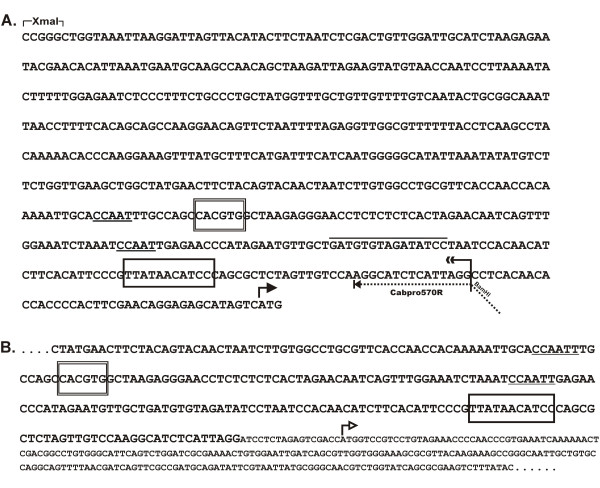
**Sequence of the Cab19 promoter region and junction with *uid*A gene**. Double strand sequence of the Cab19 promoter is shown in A. The sequence was analyzed by the PLACE program [35]. A consensus TATA box is enclosed in a single-line box; two upstream CAAT boxes are underlined; the G-box is enclosed in a double-line box; a GATA-like sequence associated with tissue-specificity is overlined. The Cabpro570R primer location is indicated along with the additional BamHI site; the double reverse arrow marks the 3' end of the Cab19 promoter generated using the Cabpro570R primer; the right-facing arrow indicates the position of the translation start site for the peach Lhcb2*Pp1 gene. B. The sequence surrounding the junction between Cab19 and *uid*A is presented. Cab19 sequence is in upper case; *uid*A is in small caps; the right-facing open arrow indicates the translation start site of the *uid*A (GUS) gene.

**Figure 2:**
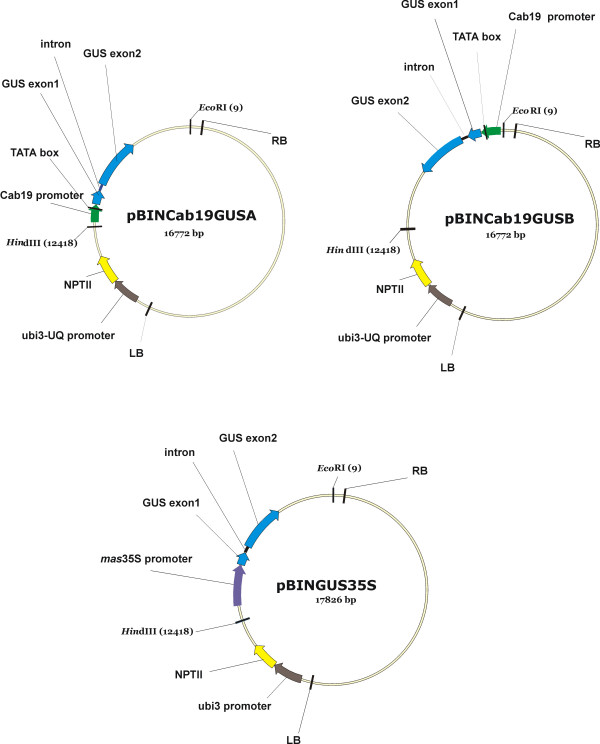
**Diagram of recombinant plasmid constructs containing the *uid*A gene under control of Cab19 or *mas*35S promoters**. Plasmids represented as closed, circular molecules are not drawn to scale (size in base pairs is indicated under plasmid name). Arrows mark positions of promoters and coding regions and indicate the direction of transcription. The positions of *Eco*RI and *Hin*dIII sites are indicated in parentheses. NPTII: neomycinphosphotransferase (kanamycin resistance); ubi3-UQ: ubiquitin promoter; LB: left border from *A*. *tumefaciens*; RB: right border from *A. tumefaciens*.

The isolation and characterization of a type II chlorophyll *a/b*-binding protein gene was described in Bassett et al. [10]. Information from the coding sequence was used to design primers for genome walking to obtain sequences further upstream of the mRNA leader. A clone containing approximately 750 bp of sequence 5' of the Lhcb2*Pp1 translation start site was isolated and sequenced. The results are shown in Figure 1A. A consensus TATA box was identified some 33 bases upstream of the putative transcription start site for Lhcb2*Pp1. Two CAAT boxes were identified upstream of the TATA box. The only other regulatory sequence noted was a single G-box element with a downstream motif containing GATA-like repeats (Figure 1A). After construction of the Cab::GUS fusion recombinant plasmids (Figure 2, See Materials and Methods), the region surrounding the junction between the Cab19 promoter and the GUS exon1 was sequenced to confirm the construction (Figure 1B).

### Histochemical analysis of transformants

A fusion product containing the GUS gene (*uid*A) under the control of the minimal Cab promoter (Figures 1B and 2) was transferred to a plant binary transformation vector (pBINPLUSARS) in both orientations relative to the left border. A control plasmid carrying GUS regulated by an enhanced *mas*35S promoter [13] was also constructed (Figure 2). All three recombinant plasmids were transformed separately into tomato 'Ailsa-Craig', and transgenic plants were recovered on antibiotic selection media. Regenerated plants were tested for the presence of *uid*A or *uid*A + Cab promoter through PCR analyses. Those regenerants that were positive for GUS or GUS + Cab were grown to maturity and maintained in a greenhouse. Ten independent lines of each construct (only 7 lines represented by the Cab::GUSA construct survived) were selected for further evaluation.

**Figure 3:**
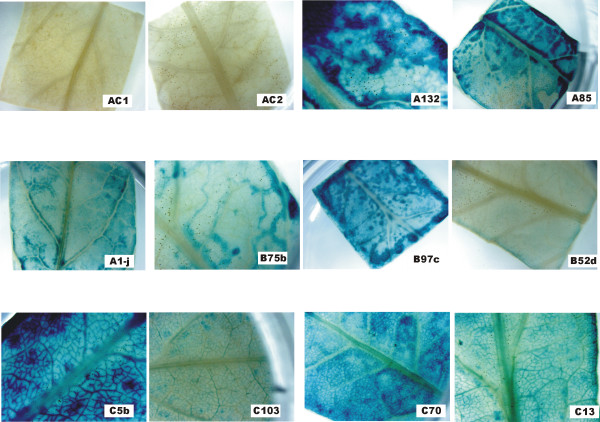
**GUS histochemical staining in leaves of transgenic tomato plants**. Expanding (30–50% full expansion) leaves of different lines of transgenic tomatoes carrying the Cab19::GUS or *mas*35S::GUS constructs were cut into ~1 cm squares and stained for GUS activity. AC1 and AC2 represent untransformed tomato lines. Independent line designations for each construct are indicated on the appropriate photograph. A lines carry pBINCab19::GUSA; B lines carry pBINCab19::GUSB; C lines carry *mas*35S::GUS. Magnification: 10×: A85 and B97c; 20×: AC1, AC2, B52d, C103 and C70; 30×: A132, A1-j, B75b, C5b and C13.

To determine the relative expression of the different lines, leaf sections from fully expanded leaves were cut and stained for GUS activity. Untransformed controls showed negligible GUS activity (Figure 3, lines AC1 and AC2), but lines carrying the recombinant constructs showed wide variation in GUS expression. Interestingly, the smaller veins in leaves from all lines carrying the *mas*35S::GUS construct stained more heavily than the lamina (Figure 3). Although this was also seen to some degree in several Cab19::GUS lines, the staining of veins in these lines was considerably less than what was observed with lines carrying the control construct. Similar results were obtained from transverse-sections through the major vein of leaves stained for GUS activity (data not shown), i.e. staining in the vascular bundles of Cab19::GUS lines was spotty compared to staining in *mas*35S::GUS lines.

**Figure 4:**
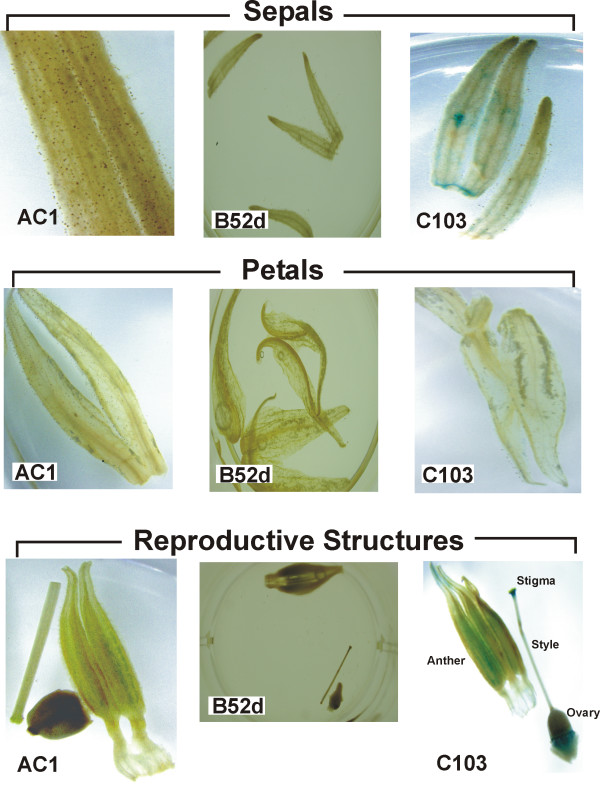
**GUS histochemical staining of flower parts**. Flowers from an untransformed control (AC1), a line carrying the pBINCab19::GUSB construct (B52d) and a line carrying *mas*35S::GUS (C103) were stained for GUS activity. Magnification: 5×: B52d sepals, petals and reproductive structures; 10×: C103 sepals, AC1, and C103 petals; 20×: AC1 sepals and reproductive structures, C103 reproductive structures.

**Figure 5:**
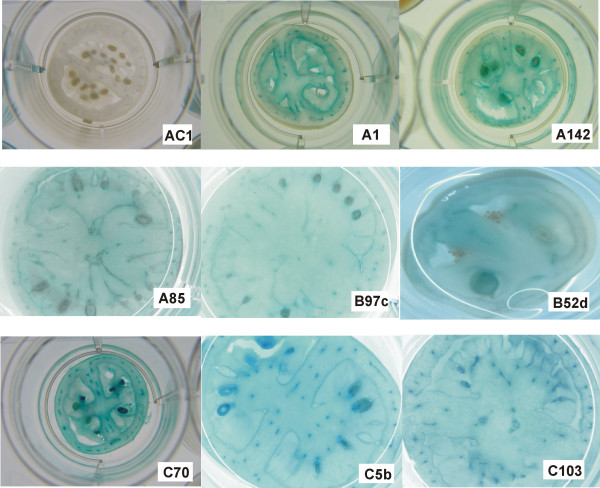
**GUS histochemical staining of green fruit**. Immature green fruit were sliced transversely and stained for GUS activity. AC1: untransformed control; lines as in Figure 3. Magnification: 2×: AC1, A1, A142 and C70; 4×: A85, B97c, B52d, C5b and C103.

**Figure 6:**
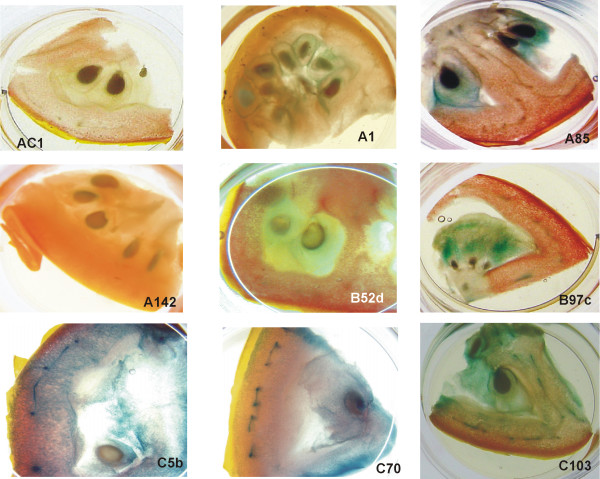
**GUS histochemical staining of red fruit**. Red (fully ripe) fruit were sliced transversely and stained for GUS activity. AC1: untransformed control; lines as in Figure 3. Magnification 2×.

Histochemical staining for GUS activity in floral parts was negative for all lines carrying Cab19::GUS in either orientation (Figure 4, illustrated for line B52d). In contrast, staining of floral structures was observed in a number of lines carrying the *mas*35S::GUS construct (Figure 4, illustrated for line C103). GUS activity was lowest in petals, higher in sepals and highest in anthers and stigmas. Immature green fruits and fully ripe (red) fruits expressed variable GUS activity (Figures 5 and 6). In green fruits, staining appeared to be more intense in vascular bundles, especially in the *mas*35S::GUS lines. In red fruits little GUS activity was observed in the Cab19::GUS lines, except for some light staining in locular material (Figure 6). Staining in the vascular bundles of the *mas*35S::GUS lines was intense, as was staining in locular tissues in these lines.

### Quantification of GUS activity

**Figure 7:**
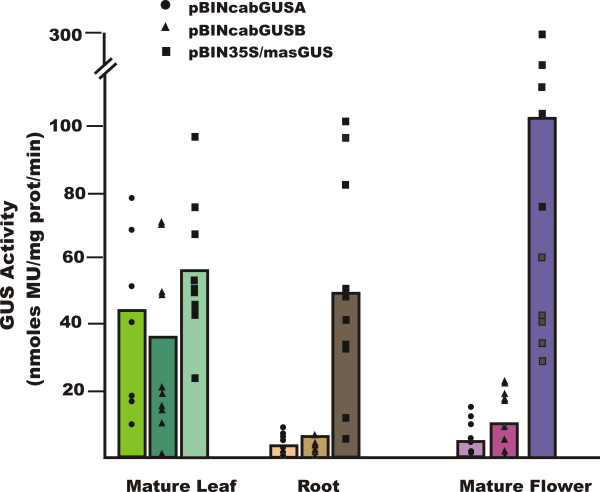
**Quantitative GUS activity in transgenic tomato vegetative and floral organs**. GUS activity was quantified in whole expanding leaves, whole flowers, and mature roots using 4-methylumbelliferone [MU] as substrate. Protein concentration was determined by the Bradford assay (Bradford 1976). Measurements were made on two reps of each independent transgenic line and an untransformed control. Activity in the untransformed control was subtracted from each transgenic sample. Ten independent B and C lines and seven independent A lines were analyzed. Each point represents a single transgenic line; the bars show the average of GUS activities for all transgenic lines of each construct.

**Figure 8:**
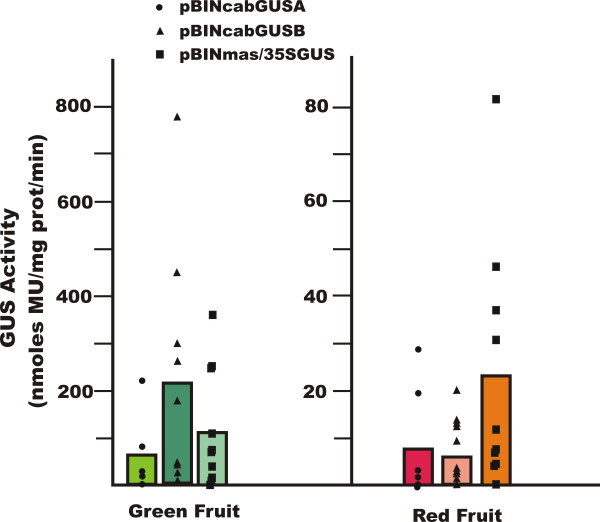
**Quantitative GUS activity in transgenic tomato fruit**. GUS activity was quantitated as before. Five whole immature green fruit and five whole fully ripe red fruit were collected and analyzed from each line. The symbols represent measurements averaged across the five fruit samples from each transgenic line; these measurements were corrected for background activity (untransformed controls) as in the legend to Figure 7. Ten independent B and C lines and seven independent A lines were analyzed. The bars show the average of GUS activities for all transgenic lines of each construct. Note the scale difference between green and red fruit.

GUS activity was quantified for all transgenic lines using the MUG assay. Activity profiles for expanding leaves, mature roots and whole flowers are shown in Figure 7. For all lines tested a range of GUS activities was seen in leaf tissue. However, in roots and flowers of transgenic lines carrying Cab19::GUS, activities were considerably lower than those seen in the *mas*35S::GUS lines, although some of the *mas*35S::GUS individual lines had low GUS activity in roots and/or flowers. GUS activity in whole fruits was also observed to vary considerably across independent transgenic lines (Figure 8). Although immature green fruits expressed relatively high levels of GUS activity (note difference in scale) in all lines, red fruit carrying a Cab19::GUS construct expressed 3–5-fold lower activity on average than fruit from lines carrying *mas*35S::GUS.

### RealTime quantification of *Cab *and GUS RNAs from light- or dark-exposed seedlings

**Figure 9:**
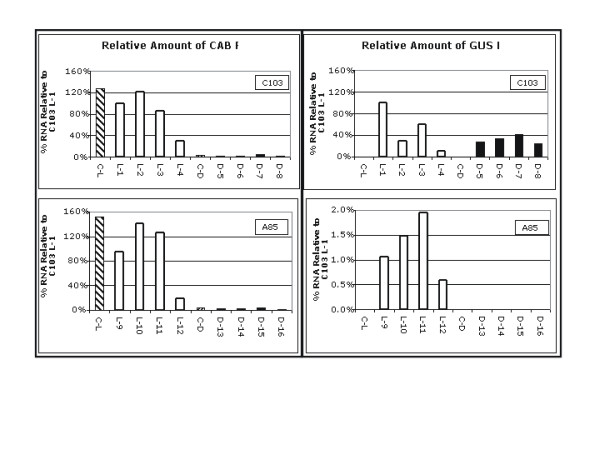
**Real time Reverse Transcription-Polymerase Chain Reaction determination of light responsiveness of Cab19**. Selfed seed from transgenic lines A85 (pBINCab19::GUSA) and C103 (pBIN*mas*35S::GUS) and the untransformed control line, AC1, were germinated in the dark. Half the germinated seedlings from each line were transferred into the light and half were kept in the dark for 48 h. RNA extracted from the epicotyls of individual seedlings that tested positive for GUS served as templates for real time Reverse Transcription-Polymerase Chain Reactions. Results are reported on a relative scale as a percentage of C103 (light exposed seedling 1:L1) for each primer pair [native tomato Cab primers and GUS primers]. C-L: AC1 control seedling exposed to 48 h light; L1-L4: four different seedlings from the C103 transformed line on plates exposed to 48 h light; L9-12: four different seedlings from the A85 transformed line on plates exposed to 48 h light; C-D: AC1 control seedling kept in the dark for 48 h; D5-D8: four different seedlings from the C103 transformed line on plates that remained in the dark for 48 h; D13-D16: four different seedlings from the A85 line on plates that remained in the dark for 48 h. Note difference in scale for GUS RNA levels in A85 seedlings.

To determine whether or not the minimal Cab19 promoter still contained light responsive element(s), Real-Time PCR was performed on seedlings from the A85 (Cab19 promoter, high GUS activity in leaves), C103 (enhanced 35S promoter, medium-high GUS activity in leaves) and AC1 (untransformed control) lines. Real-Time PCR results were shown previously to correspond with GUS activity measurements when lines varying in leaf GUS activity were analyzed (data not shown). Real-Time analyses were performed on A85, C103 and AC1 seedlings germinated in the dark and subsequently exposed to light or kept in the dark. Figure 9 presents those results demonstrating that the native tomato CAB gene is expressed at a much lower level in the dark for both lines, as expected. GUS expression in C103 was similar in light or dark while in contrast, GUS expression in A85 was completely light-dependent (Figure 9; note the scale differences of the graphs for GUS RNA).

## Discussion

### Sequence analysis of the peach Lhcb2*Pp1 gene promoter

We have described the isolation and characterization of the promoter of a peach type II chlorophyll *a/b*-binding protein gene (Lhcb2*Pp1 [Cab]). The promoter was isolated as a minimal promoter containing ca. 550 bases of sequence upstream of the Cab translation start site. Analysis of this region for *cis*-acting elements located a consensus TATA box near the 3' end of the promoter, two CAAT boxes, a single G-box sequence (CCACGTG) and a motif conserved in several promoters containing GATA-like elements. TATA boxes control basal transcription from RNA polymerase II and have been highly conserved in eukaryotes during evolution [14]. CAAT boxes located within ca. 150 bases upstream of a TATA box can enhance the basal level of transcription [15], and these elements can also be found in a variety of eukaryotes. In plants, G-box elements are responsive to a variety of hormones and other signals, including light [16], though others have shown that promoters lacking a G-box core are fully light inducible [17,18]. Hence, the G-box in the Lhcb2*Pp1 promoter may function as a light-responsive element. The last identified motif present in this Cab promoter is a GATA-containing element similar to an activation sequence binding site required for leaf-specificity in transgenic tobacco, but not light regulation [19].

### Tissue-specific regulation of the GUS reporter gene under Lhcb2*Pp1 promoter control

Constructs synthesized using the minimal Cab promoter to drive expression of GUS were used to transform tomato to assess expression. Two different orientations of the promoter with respect to the left border were tested. No significant differences were observed between two (A and B) orientations. Histochemical analysis of 27 transgenic lines (7 for pBINCab19GUSA, 10 for pBINCab19GUSB and 10 for pBIN*mas*35SGUS) indicated considerable variability in expression among individual lines. It is presumed that this variability is due in part to position effects generated from random integration of plasmid DNA into the chromosome and in part to differences in the number of integrated copies between independent lines. In fact Southern analysis suggested that higher expressing lines of all transgenics appeared to have more than one copy of the construct inserted into the genome (data not shown). Although in general higher copy number accounts for higher expression of resident genes, the correlation is not always perfect [8]. In some cases the location of an inserted sequence on the chromosome influences expression due to the presence of enhancer/suppressor elements, as well as other factors related to chromosome structure.

Nevertheless, despite the variability in expression among lines, some consistent differences were observed between Cab19::GUS constructs and the *mas*35S::GUS controls. In leaf, more GUS activity was seen in vascular tissue in the *mas*35SGUS control lines, than in the Cab19::GUS lines. These results are similar to those observed with Rubisco small subunit vs. 35S promoters in transgenic apple [8]. In addition, no GUS activity was detected in floral tissues from the Cab19::GUS lines, whereas, staining in the stigmas, anthers, sepals and petals was observed in the *mas*35S::GUS lines tested. These observations were supported by the quantitative MUG assays of GUS activity. For example, while there was substantial overlap in the range of activity seen in leaves of plants containing either the Cab19::GUS or *mas*35S::GUS constructs, the average expression for all transgenic lines indicated that Cab19::GUS lines had 60–80% of the average observed for the *mas*35S::GUS lines. Gittins et al. [8] reported similar results in transgenic apple shoots comparing 35S with the tomato RBCS3C and soybean SRS1 promoters. Yang et al. [9], however, reported activities of another photosynthetic promoter (PNZIPpro) to be ca. 5–6-fold higher than 35S::GUS. The *mas*35S::GUS activities in our study appear to be 10-fold higher in leaf than the 35S::GUS activity in the study by Yang et al. [9], after adjusting for differences in activity units between the two studies. The Q3 photosynthetic promoter construct contains 665 bases 5' of the PNZIP translation start site [9] and is equivalent in size and sequence to the Cab19 construct. Comparison of the activities of these two promoters indicates that they are virtually equivalent in leaf, root and flower tissues. In addition, unlike the PNZIP promoter, the Cab19 constructs contain no potential upstream AUGs that could interfere with translation of fused genes [20,21].

There was no overlap in GUS activities between roots and flowers expressing Cab19::GUS or *mas*35S::GUS. Both Cab19::GUS constructs expressed very low levels of GUS (5–10% of *mas*35S::GUS). These results are also consistent with the transgenic apple experiments where expression of RBCS3C::GUS or SRS1::GUS in roots was 1–4% of the 35S::GUS construct [8]. In addition, our observations support previous studies indicating that the integrated intact 35S CaMV promoter is active in most plant tissues [6].

To our knowledge, no reports examining the expression of genes under control of photosynthetic promoters in fruit have been published. Since fruit represent an economically significant portion of the agricultural production and contribute to human health and well being, it is important to evaluate promoters on their ability to restrict expression in tissues other than the edible fruit. Histochemical staining of green (very young) fruit showed that expression of GUS was nearly identical in slices from transgenic tomatoes carrying either the Cab19::GUS or *mas*35S::GUS constructs, except that in general, seeds of fruits from *mas*35::GUS transgenic plants stained more intensely. Elevated mRNA levels of another photosynthetic gene, Rubisco small subunit (RBCS1 and RBCS2), was previously reported in young fruit of tomato [22]. Furthermore, since young green tomato fruits are capable of limited photosynthesis [23], expression of Cab19::GUS in young green fruit is not surprising.

Histochemical staining of red (mature ripe) fruits carrying Cab19::GUS was higher in the locular matrix than in the mesocarp or skin. This observation is consistent with microarray analysis of early fruit development in tomato where high photosynthetic gene activity (including Cab) was observed specifically in locules [24]. Similar results were obtained during promoter analysis of the tomato RBCS3 family, showing little GUS staining in the exocarp and mesocarp, but a range from slight to intense staining in locules of mature fruit [7]. As with immature, green fruits, staining was more intense and pervasive in slices from the *mas*35S::GUS lines. The quantitative results also reflected these observations in that, although there was overlap among the lines, there were more *mas*35S::GUS lines (4/10) with activities greater than 25 nmoles 4-MU/min/mg in ripe fruit, than in the two Cab19::GUS lines (1/17).

### Light responsiveness of the Lhcb2*Pp1 promoter

That the minimal Cab19 promoter maintained its responsiveness to light is illustrated by the lack of detectable GUS mRNA in transgenic etiolated seedlings germinated and kept in the dark. Although the relative levels of GUS in transgenic Cab19::GUS epicotyls were low compared to the *mas*35S::GUS lines, the response to light was dramatic and qualitatively similar to that obtained for the native tomato cab type II gene. Based on the overall results of the GUS activity measurements and the real time RT-PCR experiments, we conclude that the minimal Cab19 promoter retains its tissue specificity and responsiveness to light. This promoter can be used for the regulation of transgenes that need to be expressed in leaves or young fruit, but do not require expression in roots or mature fruit for biological effect.

### Biotechnological applications for a leaf- and green fruit-specific promoter with minimal expression in edible roots and fruit

There are a number of biotechnological applications which require expression of a desirable gene in leaves or immature fruit of economically important plants. For example, resistant genes to control damage by insect feeding could be expressed in the primary tissue under attack and not in every tissue/organ of the plant. In this way the Cab19 promoter could be used where insects damage developing fruits either directly or by preferentially laying eggs on young fruit, as seen with plum curculio (*Conotrachelus nenuphar*) or first generation coddling moths (*Carpocapsa pomonella*). Regarding leaf-specific expression, the Cab19 promoter was recently shown to effectively drive expression of an RNAi construct in plum to control plum pox infection [25]. It is likely that the high level of expression generally obtained with the CaMV35S promoter will not be necessary to effectively express desirable genes in all cases. In those instances the Cab19 promoter will be an acceptable alternative for controlling genetically engineered resistance to pests which target leaves and green fruit.

## Conclusion

The minimal Lhcb2*Pp1 promoter (Cab19) described here can be used in heterologous expression of genes in photosynthetic tissues, including immature, green fruits. This promoter contains all of the *cis*-elements for tissue-specific expression in photosynthetically active tissues. In addition elements contributing to light regulation of this promoter are also present. Such a promoter will be useful in a variety of applications where expression of select genes in leaves and immature fruit is important, but where expression in roots, mature fruit and flowers is minimized.

## Methods

### Growth and maintenance of plant material

Seeds of an isogenic line of tomato (*Lycopersicon esculentum *Mill) cv Ailsa Craig were kindly provided by Dr. James J. Giovannoni, USDA-ARS, Plant, Soil and Nutrition Laboratory, Ithaca, NY 14853, USA. Seed germination and transformation of tomato cotyledons were done as described by Fillatti et al. [26], with modifications suggested by Dr. Jan Oakes (Calgene, Inc., Davis, CA, personal communication). Cotyledons were cut into two to three pieces, precultured on MS (Murashige & Skoog, [27]) medium containing 2 mg/L zeatin, inoculated for 15 minutes with disarmed *Agrobacterium tumefaciens *strain EHA105 containing pBINcab::GUSA, pBINcab::GUSB or pBIN*mas*35S::GUS (see below and Figure 2). The inoculated cotyledon pieces were cocultivated two to three days on MS + 2 mg/L zeatin medium, and transgenic shoots were regenerated in the same medium containing 100 mg/L kanamycin and 500 mg/L cefotaxime. Putative transgenic shoots were assayed for the presence of the peach cab promoter and *uid*A using cab primers (cab1F: 5'-GGAGAATCTCCCTTTCTGCCCTGC-3'; cab5R: 5'-CACTCCGGATTACTCTACGG-3') or GUS forward and reverse primers [28] in polymerase chain reactions [PCR]. Template genomic DNA was extracted from leaves of transgenic plants using a protocol (FastDNA) developed by Qbiogen (Irvine, CA). A DNA blot probed with a PCR-generated fragment from the *uid*A gene confirmed that select lines were independently transformed (data not shown).

Transgenic tomato lines were maintained in a greenhouse under natural light. Plants were potted in Metromix 360 soil and watered daily or as necessary. Plants were fertilized with a dilute (1/2 tbs/gal) solution of MiracleGro (Scotts Company, Maryville, OH) every 3–4 weeks. Selected lines were propagated vegetatively by rooted cuttings several times a year; cuttings were periodically checked for the presence of transgenes to insure that chimeric material was not propagated.

#### Isolation of the peach chlorophyll a/b-binding protein gene promoter

To obtain the promoter controlling the peach (*Prunus persica *L. [Batsch.]) type II cab gene, Lhcb2*Pp1 (GenBank Ac. No. AF039598; [10]), the GenomeWalker kit (BD Biosciences, Palo Alto, CA) was used. Gene-specific primer 1 was pch108D (5'-AACCCGCCAGACCCGGTATGGTATACGAAACC-3') and gene-specific primer 2 was pch108E (5'-TACGCCGGCGGTGGTTCCGGCGGTTAGAAAG-3'). The product obtained was cloned into pCR2.1 (Invitrogen, Carlsbad, CA) and both strands of the recombinant plasmid (pCab19) were sequenced to confirm the identity of the upstream cab sequence. The 623 bp promoter sequence with the first eleven amino acids of Lhcb2*Pp1 are reported in GenBank (Ac. No. EF127291).

#### Construction of promoter-GUS fusion plasmids

A minimal cab promoter was obtained as follows: A PCR product (560 bp) was generated from a genomic DNA template using the Universal AP2 primer (Genome Walker kit) and a gene-specific primer, cabpro570, containing a built-in *Bam*HI site (5'-ATGTATCAATGGATCCTAATGAGATGCC-3'). The product was excised from a 1% agarose gel, cleaned up over a GlassMax (Gibco-BRL, Bethesda, MD) column and digested with *Bam*HI and *Xma*I. The product was ligated into pUCAP [29] digested with *Bam*HI and *Xma*I to yield pCAPCab19-2. To add a fragment containing *uidA *(GUS) with the PIV2 intron [30], we used pGA482GGI digested with *Hind*III and *Sal*I. pGA482GGI was constructed as follows: 1) the *Eco*RI site was removed from pGA482G [31,32]; 2) the 5.4 kb *Hind*III-*Kpn*I fragment from pCNL56 [13] was ligated into pGA482G minus *Eco*RI; and 3) the *Eco*RI site in the PIV2 intron of GUS was removed creating pGA482GGI. The *Hind*III-*Sal*I fragment from pGA482GGI containing Exon1^GUS^-intron-Exon2^GUS^-*ocs *terminator was ligated into pCAPCab19-2 creating pClone48. Restriction endonuclease mapping of pClone48 indicated that it carried an additional ca. 6.8 kbp band of unknown sequence from pGA482GGI. To eliminate this fragment, pClone48 was digested with *Hind*III and *Sal*I, diluted 10-fold in water, heated to 70°C for 10 min, cooled to RT and self-ligated with T4 Ligase (Stratagene, La Jolla, CA) following the manufacturer's protocol. The resulting product (pClone7relig) was sequenced on both strands surrounding the two junctions and determined to contain only the GUS (Exon1-intron-Exon2-*ocs *terminator) fused to the Cab19-2 promoter. The *Kpn*I digestion fragment containing the Cab19-2-GUS fusion product from pClone7relig was ligated into *Kpn*I-digested pBINPLUSARS to create pBINCab19GUSa and pBINCab19GUSb, representing both orientations with respect to the *Pac*I-*Asc*I pBINPLUSARS MCS (Figure 2). pBINPLUSARS was a kind gift from Bill Belknap (USDA, ARS, WRRC, Albany, CA).

The *mas*35S promoter-GUS fusion product was created as follows: The *Hind*III/*Kpn*I fragment containing a sub-domain of the *mas *promoter joined to a minimal 35S promoter and fused to *uidA *in plasmid pCNL65 [13] was directionally ligated into pUCAP digested with *Hind*III and *Kpn*I to create plasmid pUCAP*mas*35SGUS. Digestion with *Asc*I and *Pac*I and ligation into pBINPLUSARS yielded pBIN*mas*35SGUS. pCNL65 was a kind gift from Dr. Stan Gelvin (Dept. of Biological Sciences, Purdue Univ.). pBIN*mas*35SGUS, pBINCab19GUSa and pBINCab19GUSb were transformed into *A. tumefaciens *EHA105 by electroporation.

#### Analysis of GUS activity

Ten independent peach Cab/GUS-positive transgenic lines for each construct were obtained; however, three lines carrying pBINCab19GUSa were lost while being maintained in the greenhouse. All PCR-positive transgenic lines were assayed for GUS expression by histochemical analysis of transverse sections from leaves at approximately 30–50% of full expansion, or whole flower parts from open flowers and roots from mature plants [3]. For histochemical analysis of fruit, transverse slices from immature green fruit (5–6 cm in circumference) and fully ripe (100% red and soft to the touch) fruit were used. Quantitative analysis of GUS activity was performed on whole organs using the MUG assay [33] with modifications described by Moon and Callahan [3]. Production of 4-methylumbelliferone [4-MU] was measured using a fluorometer (CytoFluor, Applied Biosystems, Foster City, CA). The amount of 4-MU was determined from a standard curve. Protein concentrations of the samples were determined using Bradford reagent [34] and bovine gamma globulin as a standard (Bio-Rad, Hercules, CA). GUS activity was expressed as pmol 4-MU min^-1 ^μg^-1 ^protein.

#### RNA extraction and RealTime-Polymerase Chain Reactions of seedlings germinated in light or dark

Selfed seeds collected from lines A85, C103 and AC1 (an untransformed control) were sterilized, placed on MS plates and wrapped securely in foil. Plates were kept at room temperature for 12 days. On the 12^th ^day, foil was removed from one plate and placed in the light (cool white fluorescent bulbs) for 48 h. At that time the light-exposed seedling epicotyls were harvested, followed by seedlings on plates that had remained in the dark during the additional 48 h. The dark-exposed seedlings were harvested under green light. Samples were immediately frozen at -80°C and then lyophilized.

RNA was extracted from lyophilized tissue using Trizol according to the manufacturer's protocol (Invitrogen). PCR using GUS-specific primers was performed on RNA extracts containing genomic DNA templates, and RNAs giving a negative result, i.e. assumed to be homozygous negative for *uid*A, were discarded. Contaminating DNA was removed from the selected RNAs using a DNA-Free kit (Ambion, Austin, TX) following the manufacturer's directions. Three sets of primers were used, 26S rRNA primers and UidSau primers from Moon and Callahan [3] and tomato CAB primers from this study: TomCab4AF (bp285 accession # M17558) 5'-ATTACGGATGGGACACTGGTGGAC-3' and TomCab4AR (bp404 accession # M17558) 5'-CGGGGAAAACACAACCTAAAGC-3'. RNAs were subjected to Real Time-PCR using the SYBR Green Master Mix kit and reverse transcriptase (Applied Biosystems) following the manufacturer's directions using 2 μl of RNA in a 10 μl reaction. RNA was diluted 1 to 40,000 for the 26S rRNA reactions, 1 to 200 for the tomato CAB reactions and 1 to 12 for the GUS (*uid*A) reactions. The reactions were run on an ABI7900 sequence detection instrument (Applied Biosystems) programmed to heat for 30 min at 45°C, then 10 min at 95°C, followed by 40 cycles of 1 min at 60°C and 30 s at 95°C. This was followed by a denaturation step to determine the melting point of the products formed in order to verify that a single identical product was formed. All reactions were done in triplicate and the results were averaged. Standard curves for each primer pair were derived from a dilution series, and the relative amount of RNA in each sample was determined from the standard curves. The amounts of the GUS and tomato Cab RNA were adjusted by the differences in the amount of 26S rRNA to account for any variation in the concentrations of the total RNA in each reaction. Triplicate no-template controls were included for each specific primer pair and the denaturation profiles of the no-template controls were used to determine if any product was detected.

## Abbreviations

MCS, multicloning site; Cab, chlorophyll *a/b *binding protein; PCR, polymerase chain reaction; RT, reverse transcription; GUS, β-glucuronidase; PIV2, portable intron 2 from the potato ST-LS1 gene; CaMV, cauliflower mosaic virus;

## Authors' contributions

CLB, AMC and RS conceived the idea and designed the experimental approaches. CLB and TSA isolated the cab19 promoter and made the promoter::GUS fusion constructs. RS and CS performed the transformation and regeneration of tomato plants. CLB analyzed transformants for GUS activity. AMC performed the real time RT-PCR light/dark experiment. All authors contributed appropriately to the writing of the manuscript.
